# ClipSV: improving structural variation detection by read extension, spliced alignment and tree-based decision rules

**DOI:** 10.1093/nargab/lqab003

**Published:** 2021-02-01

**Authors:** Peng Xu, Yu chen, Min Gao, Zechen Chong

**Affiliations:** Department of Genetics, the University of Alabama at Birmingham, Birmingham, AL, 35294, USA; Informatics Institute, the University of Alabama at Birmingham, Birmingham, AL 35294, USA; Department of Genetics, the University of Alabama at Birmingham, Birmingham, AL, 35294, USA; Informatics Institute, the University of Alabama at Birmingham, Birmingham, AL 35294, USA; Informatics Institute, the University of Alabama at Birmingham, Birmingham, AL 35294, USA; Department of Medicine, Division of Cardiovascular Disease, the University of Alabama at Birmingham, Birmingham, AL 35233, USA; Department of Genetics, the University of Alabama at Birmingham, Birmingham, AL, 35294, USA; Informatics Institute, the University of Alabama at Birmingham, Birmingham, AL 35294, USA

## Abstract

Structural variation (SV), which consists of genomic variation from 50 to millions of base pairs, confers considerable impacts on human diseases, complex traits and evolution. Accurately detecting SV is a fundamental step to characterize the features of individual genomes. Currently, several methods have been proposed to detect SVs using the next-generation sequencing (NGS) platform. However, due to the short length of sequencing reads and the complexity of SV content, the SV-detecting tools are still limited by low sensitivity, especially for insertion detection. In this study, we developed a novel tool, ClipSV, to improve SV discovery. ClipSV utilizes a read extension and spliced alignment approach to overcoming the limitation of read length. By reconstructing long sequences from SV-associated short reads, ClipSV discovers deletions and short insertions from the long sequence alignments. To comprehensively characterize insertions, ClipSV implements tree-based decision rules that can efficiently utilize SV-containing reads. Based on the evaluations of both simulated and real sequencing data, ClipSV exhibited an overall better performance compared to currently popular tools, especially for insertion detection. As NGS platform represents the mainstream sequencing capacity for routine genomic applications, we anticipate ClipSV will serve as an important tool for SV characterization in future genomic studies.

## INTRODUCTION

Structural variations (SVs), which refer to genomic variants over 50 bp in length, are important sources of genomic mutations and potential causes of various human diseases (1). Over 8.5 Mbp genomic sequences belong to SVs that are shared in human populations (2). For decades, many efforts have been made to discover the SV events and resolve exact breakpoints in human genomes (3). Early studies relied on array CGH and SNP microarray to detect large-scale SVs and copy number variants (4,5). With the advancement of the next-generation sequencing (NGS) platform, paired-end short reads from whole genome sequencing (WGS) experiments have been widely applied to the human genome. The NGS platform shows better resolution in identifying and characterizing short SV events at the base level (6). More recently, long reads from the third-generation sequencing platforms have been employed for SV discovery in human genome (7). The circular consensus sequencing (CCS) or HiFi sequencing technology from PacBio further improves the long-read sequencing by generating high-fidelity (accuracy 99.8%) reads (8). However, compared with the long reads sequencing platform, NGS platform still dominates the current WGS studies due to the high-base accuracy, high-throughput and low-cost advantages (3).

Based on the NGS platform, several methods have been developed to detect SV events from short reads (9). These methods can be classified into read depth, split reads, discordant read-pairs, (local) *de novo* assembly, and/or a combination of these methods (10). However, the SV detection from short reads are still limited by a low sensitivity and the detected events are biased toward deletions (8). There are two hindering factors with major influences on the performance of SV tools: the length of sequencing reads and the complexity of SV content. Currently, the read length for a typical NGS study ranges from 100 to 250 bp. Accurate alignment of the short reads onto the reference genome around the SV regions is computationally challenging (11). For the complexity of SV configuration, it was estimated that around 55% of SVs are present in repetitive sequences in human genome (2). Those repetitive regions pose a great challenge for accurate read alignment, as well as read assembly when resolving large insertion events. The complexity of SV sequences also indicates that traditional simulation analyses with random SV sequences may not properly estimate the performance of SV tools on real human genomes.

In this study, we developed a novel SV detecting tool, ClipSV, to comprehensively characterize SV events from short reads. To overcome the above limitations of short reads, ClipSV has two major improvements compared with previous methods. First, it employs read extension and spliced alignment methods to resolve read length problem. Second, it implements a tree-based decision rules to efficiently process SV-containing reads and comprehensively detect insertion events. Rather than generating simple and random SV sequences, our simulation experiments were performed by spiking real SV events, which retains the complexity nature of the human genome. Based on evaluations from both simulation and real data, ClipSV shows a better performance than the current popular tools.

## MATERIALS AND METHODS

### Detection of SV-containing reads

ClipSV implements a multithreaded pipeline to detect SV signals. By retrieving aligned reads in 1 Mb region of a chromosome, ClipSV calculates the read length from the longest aligned length. The mean and the standard deviation of the insert size are calculated with all the fragment size of primary aligned reads with a mapping score equal 60. With the read length and insert size, ClipSV collects and categorizes all kinds of reads that are not perfectly aligned: (i) clipped reads are collected based on the feature that the read has any sequence (longer than 5 bp) clipped during alignment. (ii) Split reads are collected when they contain a supplementary alignment based on the SA tag in the BAM file. (iii) Discordant read pairs are defined as when the read pairs have an insert size longer than min(mean(insert size) + sd(insert size), mean(insert size) + 300). (iv) Translocation reads are collected based on the feature that the two paired reads are aligned to different chromosomes. (v) Inversion reads are collected based on the feature that the two read pairs have the same direction during alignment. For the reads that are clipped during alignment, the base qualities were examined for the mapped region and the clipped region, respectively. The ratio of high-quality bases is calculated by dividing the number of high-quality base (Phred value > 20) by the total bases in the mapped or clipped region. All the reads with a duplication mark are filtered out before downstream analysis.

### Read extension and spliced alignment

**Figure 1.**
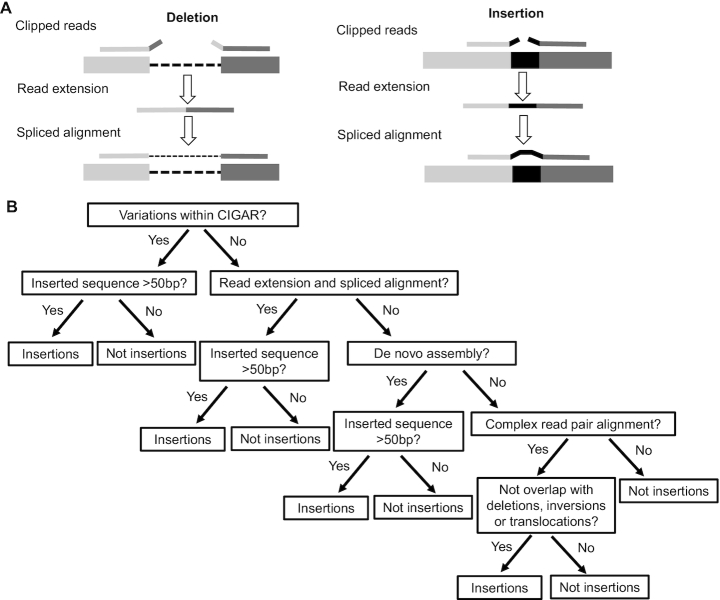
Overview of the key methods implemented by ClipSV. (**A**) Schematic diagram of the read extension and spliced alignment to detect SVs. In the process of read extension, ClipSV slides along the chromosome and dynamically concatenates the clipped reads within 1 kb bin based on the reciprocal overlapping of the last 30 bp regions. With the reconstructed long sequences and the raw clipped reads, ClipSV utilizes the spliced alignment method to detect SV events (left: deletions; right: insertions). (**B**) The pipeline of the tree-based decision rules for insertion detection. By extracting all the positions supported by the clipped reads, ClipSV tests whether an insertion could be identified by the following decision nodes: the CIGAR string, the read extension and spliced alignment, the local *de novo* assembly, and the complex read alignment. These methods are sequentially arranged as the major decision nodes. The ‘yes’ or ‘no’ indicates whether an insertion from the SV containing position could be detected by the corresponding method. The test will terminate if an insertion event could be identified by the earlier node.

ClipSV employs read extension and spliced alignment to detect deletions and small insertions (Figure 1A). To perform read extension from the clipped reads, ClipSV filters out the clipped reads with low quality (mapping score <20, supplementary alignments). Next, it slides along the chromosome and dynamically classifies the clipped reads within 1 kb bin into two groups based on directions of their clipped sequences: the MS group and SM group (M: Matched sequence; S: clipped sequences). For each clipped read in the SM group, ClipSV iteratively checks its overlapping with the clipped reads in the MS group within 1 kb. This process compares the first n bases in the SM read with the last n bases in the MS read, starting from the largest *n* = read length −30. For each iteration, the program decreases the n value until it identifies the longest *n* bases that are shared by the two clipped reads. If *n* is >30, it will output the extended sequences by concatenating the two clipped reads. Considering potential sequencing errors, three bases shift is allowed at the boundary of the clipped reads. The resulted long sequences are reported in a FASTA format for downstream spliced alignment.

The inputs of ClipSV include two sets of reads for spliced alignment: (i) the raw clipped reads without read extension; (ii) the extended sequences from clipped reads. The spliced function in Minimap2 (v2.14) is employed to perform the spliced alignment (Li, (12)). The resulted SAM file is analyzed to identify the reads with spliced alignment in the genome. With the SAM file, the CIGAR string is extracted and analyzed to search the location with ‘N’ or ‘D’ for deletions and ‘I’ for insertions. The deletions and short insertions are identified with at least X reads support (X is set as 10% of the sequencing coverage). The low-quality alignments are filtered out before downstream analysis: (i) mapping quality is lower than 10. (ii) Boundary sequence is clipped during alignment. (iii) Aligned boundary sequence is shorter than 20. (iv) More than five regions within a read are separately aligned.

### Detection of insertions by tree-based decision rules

To detect insertions, ClipSV employs tree-based decision rules to efficiently process the clipped reads (Figure 1B). ClipSV extracts all the positions that are supported by the raw clipped reads. Then ClipSV tests whether an insertion can be discovered by the method in each node. The order of each decision tree node was arranged based on the following considerations: The CIGAR can discover indels (<50 bp); the read extension and spliced alignment can identify short insertions; the local assembly can identify large insertions and the insertions missed by spliced alignment; the complex read alignment can rescue insertions that fail to be identified by the above methods.

In detail, the positions of SV signals are collected based on the support of the raw clipped reads (5 bp region with at least X clipped reads; X is set as 10% of the sequencing coverage). Then ClipSV implements the decision tree by testing whether the SV signal could be resolved with an insertion event:

- Starting from the original SAM file, the CIGAR string is extracted to check whether SV events are within the aligned reads.
- The raw clipped reads are subjected to read extension and spliced alignment to identify short insertions.
- If an insertion fails to be identified at the location, the local assembly will be performed by extracting all the raw short reads around the breakpoint regions. Around the 1.2 kb region of a high-confident breakpoint, all the clipped reads and improperly paired reads lacking alignment flag 0 × 2 (mapping score ≥ 20), as well as their read pairs are extracted from the BAM file. Then the retrieved read pairs are converted to FASTQ format. Velvet (v1.2) is employed to perform local de novo assembly (13). Three *k*-mers are used for *de novo* assembly: 41, 61 and 81 bp. The assembled contigs are pooled together as FASTA format and aligned to the genome by Minimap2. The aligned SAM file is processed to identify two types of insertions: the fully assembled insertions and the partially assembled insertions. The fully assembled insertions are identified by ‘I’ symbol in the CIGAR string around the breakpoint region. The partially assembled events are identified by the clipped sequences (>50bp) around the breakpoint region.
- After *de novo* assembly, additional insertion events are identified by complex read alignments. Because of the sequence complexity, reads arising from insertion events are often unmapped or misaligned to other regions. To resolve such insertion events, the breakpoints of the SV events are identified with the following criteria: (a) the clipped reads are aligned with high confidence (mapping score ≥ 50, the ratio of high-quality bases ≥ 0.8), while their mates are aligned to other chromosomes or unmapped. (b) The breakpoints are supported with at least X clipped reads (X is set as 10% of the sequencing coverage). (c) The breakpoints do not overlap with indels, inversions or translocation events.

Both translocation reads and complex read alignments from insertions include clipped reads and their mates mapped to different chromosomes. However, the complex read alignments include more types of read alignments: (i) when the inserted sequences are distinct from the genome sequence (e.g. *de novo* sequence insertion), the clipped reads will be only mapped to the breakpoint regions. Meanwhile, the clipped reads in translocations need to be mapped to two different chromosome locations; (ii) when the inserted sequences are slightly similar to the genome sequence, the inserted sequence may be mapped to other chromosomes, but with a low mapping quality. Such low mapping quality will not meet the criteria to support a translocation event (mapping quality >20 for both locations). Therefore, these clipped reads in insertion regions can be distinguished from translocation reads; (iii) in the cases when the clipped sequences are quite short, only one region of the clipped reads can be mapped to the genome, leaving the clipped sequences unmapped. This kind of clipped reads will be regarded as complex read alignments to identify insertions, but they do not belong to translocation reads. To exclude the potential false positive signals, ClipSV performs an additional filtering step to ensure that translocations and other SV events are absent from the insertion positions. Consistent with long read studies that consider tandem duplications are one type of insertion events (2,8), ClipSV does not distinguish tandem duplications with the insertion events. The tandem duplication events are regarded as insertions when evaluating different SV tools.

### Detection of inversions and translocations

ClipSV relies on raw short reads to detect the inversion and translocation events. Inversion events are identified based on the reversion features from the split reads and discordant read pairs (Supplementary Figure S1). For split reads, the orientations of the primary and supplementary alignments from the split reads are analyzed. If the two alignments from one split read have opposite orientations, an inversion spanning the two breakpoints will be identified. For discordant read pairs, if the orientations of the aligned read pairs are the same, this read pair will support a large inversion event. In this scenario, the precise location of the inversion breakpoint can be inferred based on the observed and the predicted insert size of the read pair. If the two breakpoint regions contain any clipped reads, the exact breakpoints of the clipped reads will be used to estimate the positions of inversion events. If the breakpoints are supported with at least X coverage of the split reads or discordant read pairs (X is set as 10% of the sequencing coverage), the inversion will be considered as a confident event.

Translocation events involve the sequences between two chromosomes. Both clipped reads and discordant read pairs can be observed around the translocation regions (Supplementary Figure S2). For split reads, the breakpoints of the two alignments are regarded as the translocation site. For discordant read pairs, the translocation sites could be approximated by the alignments of the two read pairs. If the region of discordant read pairs overlaps with split reads, the exact breakpoints of the split reads will be used to estimate the positions of translocations. If the breakpoints are supported by at least 2*X coverage with the total number of split reads and discordant read pairs (X is set as 10% of the sequencing coverage), the translocation will be considered as a confident event.

### Simulation of the SV-containing genome and the sequencing reads

The SV events are enriched with repetitive sequences and are more prone to be distributed in centromeres and telomere regions (2). Therefore, simulating SV events that can reflect SV sequence features and chromosome locations is important to benchmark the performance of SV detection tools. To achieve this, we employed a non-random simulation strategy to simulate SV events in the genome. In this pipeline, the diploid genome was simulated separately by merging two haploid genomes. In each haploid genome, we spiked in the real deletions and insertion events of HG002 from GIAB database. These SV callsets were obtained from GIAB database (ftp://ftp-trace.ncbi.nlm.nih.gov/ReferenceSamples/giab/data/AshkenazimTrio/analysis/ NIST_SVs_Integration_v0.6/), and only ‘PASS’ events were used for simulation. These SV events were identified by integration of multiple sequencing platforms and have a high-confidence based on the evaluation of CCS reads (8,14).

Because the SV callset of HG002 in GIAB does not contain inversion and translocations, these two SV types were generated by random simulation. In real human genomes, the inversions and translocations are very few based on the evaluation of long read sequencing results (2). Therefore, 200 inversion events and 60 translocation events were simulated to benchmark performance of SV tools. To simulate inversion events, a genomic location was randomly selected while avoiding the co-occurrence with previous SV events within 1 kb region. The length of inversion was randomly assigned within 50–10 kb length. To generate translocation events, the chromosomes in the genome were grouped into two haplotypes. For each haplotype, two chromosomes were randomly selected as a pair for translocations. In total, 10 chromosome pairs were generated from each haplotype of the genome. For each chromosome pair, three reciprocal translocation events were created along the two chromosomes. Each reciprocal translocation generated two breakpoints, which lead to 60 translocation breakpoints for each haplotype. Deletions, insertions and inversions were randomly assigned to one haploid genome (heterozygous) or two haploid genomes (homozygous). Specifically, three numbers 1, 2 and 3 were randomly generated. These numbers represent three genotypes ‘1|1’, ‘0|1’ and ‘1|0’, and were randomly assigned to each SV event. Theoretically, the ratio of homozygous to heterozygous genotype in the simulation is 1:2 = 0.5. Using this method, we spiked in GIAB calls and generated 4089 ‘1|1’, 4165 ‘0|1’ and 4113 ‘1|0’ SV events. Translocation events were heterozygous and randomly assigned to a haplotype.

After simulating SV events in the genome, two haploid genome sequences were obtained. Each haploid genome FASTA file was used to simulate reads by DWGSIM (v0.1.11) (https://github.com/nh13/DWGSIM) with parameters: -C 15 -S 2 -e 0.002 -E 0.002. Then the simulated raw reads from each genome were pooled together to generate a 30× coverage of paired-end reads. Two datasets were simulated with both 150 and 250 bp read length, respectively.

The simulated reads and real sequencing reads were aligned to genome reference hg19 by BWA-MEM (v0.7.17) (15). SAMBLASTER (v0.1.24) was used to mark duplicate reads in the alignment (16). The commands used to generate the aligned bam file is: bwa mem -t 12 -R ‘@RG\tID:foo\tSM:bar’ genome.fa sim.read1.fastq sim.read2.fastq |samblaster | samtools view -1 - >out.bam. Then the aligned bam file was sorted, indexed, and used for downstream SV calling.

### Evaluation of SV signal detection by different read alignments

For the split reads and clipped reads, we extracted the aligned reads and calculated the breakpoint position. Then high-confident breakpoints, which were supported by breakpoint sites of two reads within 5 bp, were used to overlap with the high-confident SV events from GIAB database. If the breakpoint region overlapped with SV events within 200 bp window, the SV event signal was considered to be captured by this type of read alignment. For the discordant read-pairs, mate unmapped or mapped to other chromosomes, we approximated the breakpoint sites using the 200 bp boundary from start and the end positions of the mapped reads respectively. If these breakpoint sites were supported by two reads within 200 bp, they were considered as high-confident breakpoint sites. If the high-confident breakpoint sites overlapped with SV events within 600 bp window, the SV event signal was considered to be captured by this type of read alignment. Overall, in this process analyzed the captured SVsignal by different read alignments without resolving SV events.

### Evaluation of different SV tools by simulation

To evaluate performance of each tool by simulation, Lumpy (v0.2.13) (Layer *et al.*, (17), Manta (v1.3.2) (Chen *et al.*, (18) and ClipSV were used to detect SV events with default parameters. For simulation studies, the identified SV events by each tool were compared with the ground truth events. For deletions and inversions, the identified true SV events were defined as the events overlapping with ground truth with 200 bp window shift, and less than 50% of size difference. For insertions, the identified true events were defined as 200 bp shift around the breakpoint of ground truth, and <50% of size change. For large insertion events that do not have an event size, the evaluation was performed based on 200 bp shift around the breakpoint of ground truth. For translocations, the true events were defined as the events overlapping with ground truth with 200 bp window shift. The sensitivity was calculated by dividing the number of true positive events by the number of ground true events. The precision was calculated by dividing the number of true positive events by the number of total identified events. The F1 score was calculated as: 2*(sensitivity*precision)/(sensitivity+precision).

### Evaluation of different SV tools by real sequencing data

To evaluate performance of different SV tools in real genome sequencing reads, we obtained 2 × 150 bp and 2 × 250 bp reads sequenced by Illumina platform from GIAB (ftp://ftp-trace.ncbi.nlm.nih.gov/ReferenceSamples/giab/data/AshkenazimTrio/HG002_NA24385_son/). The raw reads were mapped to hg19 reference genome and down-sampled to 30× coverage. Lumpy (v0.2.13), Manta (v1.3.2) and ClipSV were run with default parameters to detect SVs.

We used two datasets to evaluate the performance of different SV tools: the high-confident SVs in GIAB database and the CCS detected SV results. As the GIAB high-confident callset only contains half of the SV events identified by the CCS dataset (8), this callset is only suitable to evaluate the sensitivity of SV tools. The SV events detected by the CCS reads was used to evaluate the precision of different SV tools. For the high-confident SVs in GIAB database, we compared the SV events with those detected by each tool. The true SV events were defined with the same criteria as the simulation analysis.

For CCS read analysis, we downloaded the mapped CCS reads from GIAB database (ftp://ftp-trace.ncbi.nlm.nih.gov/ReferenceSamples/giab/data/AshkenazimTrio/HG002_NA24385_son/). Each CIGAR string of the read alignment was analyzed. The CIGAR character ‘I’ and ‘D’ was searched by a custom python script to identify SV events within the CIGAR string. The split information was also considered to identify large deletions and insertions. The SV events supported by at least three CCS reads were considered as confident events. These SVs were used as ground truth to evaluate performance of each SV tool. The criteria were similar with those in the simulation analysis: for deletions, the identified true SV events were defined as the events overlapping with ground truth with 200 bp window shift, and less than 50% of size difference; for insertions, the identified true events were defined as 200 bp shift around the breakpoint of ground truth and <50% of size change; for the insertion events without an event size, the evaluation was only based on 200 bp shift around the breakpoint of ground truth.

## RESULTS AND DISCUSSIONS

### Methods of ClipSV to detect SV events

In human genomes, deletions and insertions represent the major types of SV events. Split reads, which have two alignments at the boundaries of the deleted sequences, are important signal for deletion discovery (Tattini *et al.*, (10). However, due to the repetitive nature of the contents of the SV events, the intrinsic short length of the sequencing reads and the uneven read distribution, the split read signal may disappear around the SV boundary (Supplementary Figure S3). Unlike split reads methods that use two alignments of a split read, ClipSV starts from all clipped reads (the reads with sequence clipped during alignment) that contain SV signals. Two key methods distinguish ClipSV from other tools. First, ClipSV employs the read extension and spliced alignment to detect deletions and short insertions (Figure 1A). Second, it adopts tree-based decision rules to comprehensively detect and classify insertion events (Figure 1B).

To alleviate the read length limitation, ClipSV seeks to extend the read length to generate long sequences and aligning the reconstructed long sequences onto the genome (Figure 1A). It first performs pair-wise overlapping of the clipped reads to generate long sequences. This process involves concatenating the two reads that contain common sequences (>30 bp) with a ‘head-to-tail’ structures between them. With the reconstructed long sequences as well as raw clipped reads, ClipSV utilizes spliced alignment method to detect SV events. Spliced alignment was originally proposed as an algorithm to construct exon assemblies from the genome background (19). Recently, it was used in mapping the long reads onto the genome reference (12). Here, taking the advantage of longer read length, spliced alignment method is employed by ClipSV to discover SV events within the reconstructed long sequences. The design of read extension and spliced alignment method has the following advantages. First, as read extension performs pair-wise overlapping of clipped reads, the computational process is efficient and the resulted long sequences are accurate. Second, unlike assembly based methods that usually reply on reads with a high coverage and try to calculate a long consensus sequence representing that region (20), the read extension method faithfully keeps useful and informative reads and the extended sequences can be treated as a single long read to support an SV event. Third, spliced alignment can identify deletions and short insertions by aligning the clipped reads to the boundary sequences of SV events. Meanwhile, the long sequences from read extension can be directly used by spliced alignment, alleviating read length limitation of the raw clipped reads.

Detecting insertion events is intrinsically difficult for short reads. Based on recent estimates from Pacbio sequencing results, the sensitivity of SV detection in current tools is still very low and biased toward deletions (8). To improve insertion detection, ClipSV implements tree-based decision rules to efficiently stream the clipped reads for insertion detection. Briefly, it first collects the SV candidate breakpoints based on the raw clipped reads. Then it implements read extension and spliced alignment to detect short insertion events within the raw clipped reads and extended long sequences. If an insertion fails to be identified at the location, the de bruijn graph based local assembly will be performed by extracting all the raw short reads around the breakpoint regions. If both spliced alignment and local assembly fail to identify the insertion event, ClipSV will test whether an insertion could be supported by the complex read alignments. Considering complex read alignments may be introduced by clipped reads of other types of SVs, ClipSV further filters the positions overlapping with deletions, inversions and translocations.

### Evaluation of SV signals captured by ClipSV

**Figure 2.**
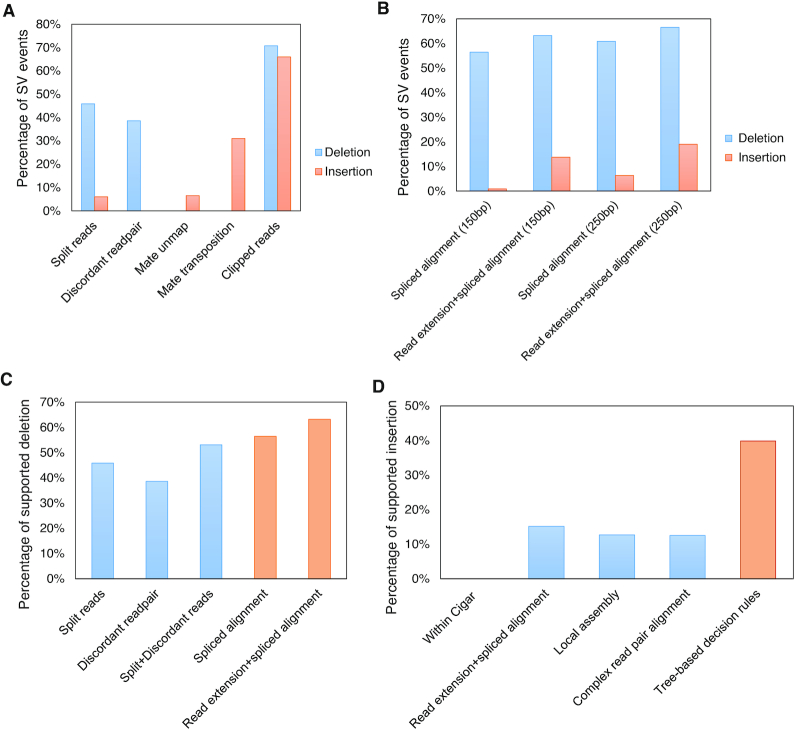
Evaluation of SV detecting methods implemented by ClipSV. (**A**) Percentage of SV events overlapping with signals of clipped reads, split reads and discordant read pairs. The Mate unmap and Mate transposition refer to the reads whose mates were unmapped or mapped to different chromosomes. (**B**) Percentage of detected SVs by read extension and spliced alignment with 150 and 250 bp read length. (**C**) Percentage of identified deletions by different methods with split reads, discordant read pairs and clipped reads. The orange column indicates the spliced alignment and read extension applied by ClipSV. (**D**) Percentage of identified insertions by different methods applied in tree-based decision rules. The orange column indicates the assembled decision tree approach applied by ClipSV. For above evaluation analyses, the high-confident SVs from chromosome 1 of HG002 sample in the GIAB database were used as ground truth. The read alignments are from 2 × 150 bp read length with 30× sequencing depth. Two reads were used to support each read signal.

Clipped reads are the starting point for ClipSV pipeline. We evaluated the performance of clipped reads in capturing SV signals. We benchmarked the results with high-confident SV events from the HG002 sample of the GIAB database (14). To compare different types of read alignments, we categorized the sequencing reads into five groups: (i) the split reads; (ii) the discordant read pairs (distance of a read pair longer than expected); (iii) the mates of read pairs unmapped; (iv) the mates of read pairs mapped to other chromosomes; (v) the clipped reads. Among 386 high-confident deletions and 567 high-confident insertions (including tandem duplications) evaluated, the split reads overlapped with 177 (45.9%) deletions and 34 (6.0%) insertions, and the discordant read pairs overlapped with 149 (38.6%) deletions (Figure 2A). Noticeably, the split reads and discordant read pairs had a bias toward deletions compared with insertions. On the other hand, 7% of insertion signals overlapped with the reads whose mates were unmapped, indicating these events are novel sequence insertions. Moreover, 31% of insertion signals overlapped with the reads whose mates were mapped to different chromosomes, suggesting the inserted sequences have sequence similarity to other genomic regions. Among the five different alignments, the clipped reads captured the most comprehensive SV signals, which included 273 (70.7%) deletions and 374 (66.0%) insertions (Figure 2A).

### Evaluation of read extension and spliced alignment methods

Read length is a key factor that influences read alignment and SV detection (7). With the advancement of short read sequencing technology, 150 bp read length is widely adopted in genome sequencing projects. Meanwhile, 250 bp read length is nowadays also available in MiSeq and NovaSeq 6000 sequencers on Illumina platform (https://www.illumina.com/). Therefore, we investigated the performance of read extension and splice alignment with different read lengths. Among the same evaluation SVs from the HG002 of the GIAB database, spliced alignment of clipped reads detected 218 (56.5%) deletions and 5 (0.9%) insertions with 2 × 150 bp sequencing reads (Figure 2B). After the procedure of read extension, it discovered 244 (63.2%) deletions and 78 (13.8%) insertions. In contrast, split reads alone detected 177 (45.9%) deletions (Figure 2C). Even when combining with discordant read pairs, it could only detect 205 (53%) deletions. This result showed the read extension and spliced alignment had a better performance than split reads and discordant read pairs in detecting deletions. For 2 × 250 bp reads, spliced alignment of clipped reads could identify 235 (60.9%) deletions and 36 (6.3%) insertions. After read extension, it discovered 257 (66.6%) and 108 (19.0%) insertions. This result showed the read extension and spliced alignment method had an even better performance with a 250 bp sequencing length.

### Evaluation of tree-based decision rules

As spliced alignment compares the two parts of clipped reads with the boundary sequences of the deletions, the length of deleted sequence is not a constraint for deletion detection. For insertions, the spliced alignment can only identify small insertion events within the clipped reads and the extended long sequences (Supplementary figure S4). To improve insertion discovery, ClipSV implements the tree-based decision rules that sequentially integrate multiple detection methods. Thus, we evaluated the performance of each detection method and their contributions to the whole pipeline. Among the evaluated SVs from the GIAB database, the read extension and spliced alignment detected 86 insertions (15%) (Figure 2D). Then these SV sites were excluded from the candidate breakpoints, and local de novo assembly was performed by extracting all the raw clipped reads. In this process, the local assembly detected 72 (13%) insertions. Next, we excluded candidate breakpoints overlapping with identified deletions, inversions and translocations. For the remaining candidate breakpoints, 71 (13%) insertions were supported by evidences of complex read pair alignments. When integrated with different methods as decision nodes, the tree-based decision rules could identify 226 (40%) insertion events. This result showed the tree-based decision rules improved the insertion detection by taking advantage of each detection method.

### Performance of ClipSV in 150 bp WGS datasets

To evaluate the overall performance at the whole genome level, we compared the ClipSV with currently popular SV tools Lumpy and Manta. These two tools were chosen because they are representative for the different methods to detect SVs: Lumpy discovers SV events by integrating SV signals from split reads, discordant read pairs and read depths (17), while Manta improves SV detection by resolving breakend graphs with a local assembly approach (18). Based on a recent study, both Lumpy and Manta were ranked among top tools for SV detection (21).

**Figure 3.**
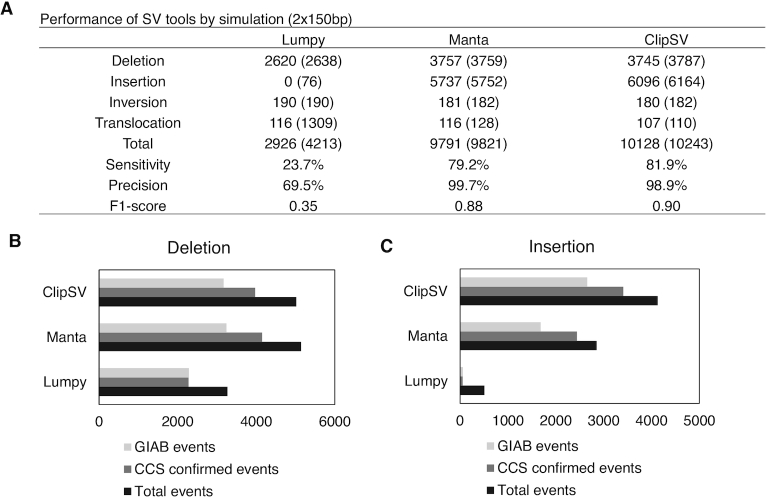
Performance of different SV detecting tools with 2 × 150 bp sequencing reads. (**A**) Performance of SV detecting tools in 2 × 150 bp simulation dataset with 30× sequencing depth. The insertions and deletions were generated by spiking in 12 047 high-confident SV events of the sample HG002 from the GIAB. The inversions and translocations were generated by random simulation. The number of true SV events were shown for each SV category, followed by the number of total detected events in brackets. Evaluation of SV tools was based on the detected SV events in each type. (**B**) Evaluation of deletion detection by different SV tools on real WGS data. Bar plots show the deletion events detected by each tool with 2 × 150 bp sequencing reads. Two datasets were used to evaluate tool performance: the GIAB high-confident SVs (light gray bars) and the CCS SVs (gray bars). The black bar indicates total detected events. (**C**) Evaluation of insertion detection by different SV tools on real WGS data.

We first constructed a nonrandom simulation dataset by spiking in 12 047 high-confident SV events of the sample HG002 from the GIAB. These SV events include 5192 deletions and 6855 insertions. We also randomly simulated 200 inversions and 120 translocation breakpoints in the genome to encompass diverse SV types. With the SV containing genome sequence, 150 bp paired-end reads were simulated to a 30× sequencing depth. Figure 3A shows the overall performance of different tools in detecting SV events in the 150 bp simulation dataset. For deletions, Manta and ClipSV had a comparable performance (3757 versus 3745, Supplementary Table S1). But Lumpy only detected 2620 events, which was much lower than Manta and ClipSV. For insertions, ClipSV had the best performance among the three tools. It detected 6069 (88.9%) insertion events. In contrast, Manta detected 5737 (83.7%) insertions and Lumpy did not detect any true insertions. Instead, Lumpy reported a great proportion of false positive events in translocations (Figure 3A). This is likely because Lumpy does not perform well in resolving insertions from translocations. For inversions, Lumpy has the best performance among the three tools. To estimate the overall performance, we calculated the F1 score for the three tools. ClipSV has a highest F1 score (0.90), followed by Manta (0.88) and Lumpy (0.35). Apart from Lumpy and Manta, we also evaluated the performance of a local assembly based SV tool svABA, which relies on String Graph Assembler and is efficient for SV detection at genome-wide scale with low memory and computing requirement (22). As svABA only outputs breakpoints without resolving SV types, we used all the breakpoints it identified to support the true SV events in simulation. The results showed that svABA had 24.6% sensitivity and 29.3% precision to detect the spiked-in SV events (Supplementary Table S2). In contrast, ClipSV showed 81.9% sensitivity and 98.9% precision (Figure 3A). These results suggest a better performance of ClipSV compared with the pure assembly based method.

We also evaluated the performance of ClipSV on the bias of the homologous SV events. Of all the simulated SV events (4089 homozygous events and 8278 heterozygous events), ClipSV identified 3493 (85.4%) of the homologous SVs and 6635 (80.2%) heterozygous events. Among the simulated deletions, ClipSV detected 1314 (76.1%) homologous events and 2431 (70.1%) heterozygous events. For insertions, ClipSV detected 2121 (92.3%) homologous events and 3975 (87.2%) heterozygous events. These results showed ClipSV has a slight bias in the detecting homologous events which are supported by more read alignment signals.

To evaluate the performance of SV tools on real WGS datasets, we analyzed 150 bp paired-end reads with 30× sequencing depth in the HG002 sample. We used the GIAB high-confident SV callset and CCS long reads to evaluate the performance of different tools. In total, ClipSV detected 9149 SV events, which included 5019 deletions and 4130 insertions, respectively. Among them, 5826 events (63.7%) are supported by GIAB high-confident SV callset and 7383 events (80.7%) are supported by CCS reads (Figure 3B and C). In contrast, Manta detected 7993 SV events, including 5141 deletions and 2852 insertions, respectively. Among them, 4927 events (61.6%) were supported by GIAB high-confident SV callset and 6590 events (82.4%) were supported by CCS reads. Lumpy reported 3777 SV events that included 3269 deletions and 508 insertions/duplications. Among them, 2348 (62.2%) and 2332 (61.7%) events were supported by GIAB high-confident SV callset and CCS reads, respectively. With CCS reads as a benchmark, ClipSV identified the highest number of true SV events (7383), followed by Manta (6590) and Lumpy (2332). Consistent with the simulation results, ClipSV had a comparable performance with Manta in detecting deletions but exhibited a significant improvement in insertion detections.

### Performance of ClipSV in 250 bp WGS datasets

**Figure 4.**
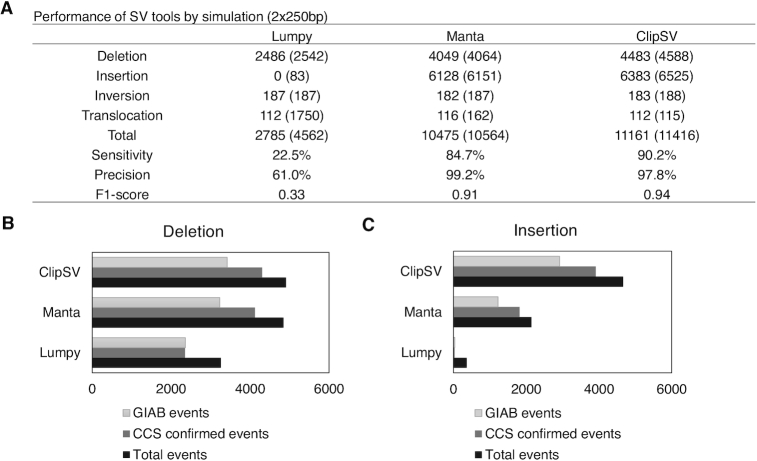
Performance of different SV detecting tools with 2 × 250 bp sequencing reads. (**A**) Performance of SV detecting tools in 2 × 250 bp simulation dataset with 30× sequencing depth. The insertions and deletions were generated by spiking in 12 047 high-confident SV events of the sample HG002 from the GIAB. The inversions and translocations were generated by random simulation. Evaluation of SV tools was based on the detected SV events in each type. (**B**) Evaluation of deletion detection by different SV tools on real WGS data. Bar plots show the deletion events detected by each tool with 2 × 250 bp sequencing reads. Two datasets were used to evaluate tool performance: the GIAB high-confident SVs (light gray bars) and the CCS SVs (gray bars). The black bar indicates total detected events. (**C**) Evaluation of insertion detection by different SV tools on real WGS data.

Using a similar strategy to 150 bp reads, we compared the performance of different SV tools with 250 bp sequencing reads. First, we spiked the same SV events into the genome and simulated 250 bp paired-end reads to a 30× sequencing depth. Compared with 150 bp simulation results (Figure 3A), both ClipSV and Manta had an improved performance with 250 bp reads. The overall sensitivity of ClipSV increased from 81.9 to 90.2% (Figure 4A). Similarly, the sensitivity of Manta increased from 79.2 to 84.7%. However, Lumpy showed a decreased sensitivity from 23.7 to 22.5%. With 250 bp sequencing reads, ClipSV excelled Manta in all kinds of SV types, including deletions, insertions, inversions and translocations (Supplementary Table S3). Consistently, ClipSV had a highest F1 score (0.94) among three tools, followed by Manta (0.91) and Lumpy (0.33). As shown in Figure 4A, compared with 150 bp sequencing reads, Manta had a higher false positive rate (28.4 versus 9.3%) in detecting translocations with a longer read length. We also evaluated the performance of svABA with the 250 bp reads. The result showed that svABA had 24.6% sensitivity and 35.1% precision to detect the spiked-in SV events, slightly better than the 150 bp reads (Supplementary Table S2).

The detection bias toward the homologous SV events was also evaluated for ClipSV. Of all the simulated SV events (4089 homozygous events and 8278 heterozygous events), ClipSV identified 3757 (91.2%) of the homologous SVs and 7404 (89.4%) heterozygous events. Among the simulated deletions, ClipSV detected 1547 (89.6%) homologous events and 2936 (84.7%) heterozygous events. For insertions, ClipSV detected 2151 (93.6%) homologous events and 4232 (92.9%) heterozygous events. These results showed ClipSV with the 2 × 250 bp reads has a better performance in detecting heterozygous events compared with the 2 × 150 bp reads.

We also investigated the performance of SV tools on real WGS datasets with 250 bp paired-end reads in the HG002 sample. Similar to the 150 bp dataset, the performance was evaluated by the GIAB high-confident SV callset and CCS long reads. ClipSV detected 9573 SV events with 250 bp reads, including 4909 deletions and 4664 insertions, respectively. Among them, 6338 events (66.2%) were supported by GIAB high-confident SV callset and 8215 events (85.8%) were supported by CCS reads (Figure 4B and C). Compared with 150 bp sequencing reads, ClipSV showed an improved performance in both deletions and insertions with 250 bp reads. In contrast, Manta totally detected 6994 SV events, which included 4848 deletions and 2146 insertions, respectively. Among them, 4457 events (63.7%) were supported by GIAB high-confident SV callset and 5938 events (84.9%) were supported by CCS reads. Compared with 150 bp sequencing reads, Manta showed a comparable performance in detecting deletions but a decreased performance in detecting insertions with 250 bp reads. For Lumpy, it totally reported 3624 SV events, including 3262 deletions and 362 insertions/duplications. Among them, 2398 (66.2%) and 2382 (65.7%) events were supported by GIAB high-confident SV callset and CCS reads, respectively. Its performance in 250 bp reads was similar to the 150 bp sequencing reads. With CCS reads as a benchmark, ClipSV identified the highest number of true SV events (8215), followed by Manta (5938) and Lumpy (2382). Consistent with simulation results, ClipSV had a better performance than Manta and Lumpy in both deletion and insertion events.

### Read depth, running time and memory cost

Finally, we evaluate the performance of ClipSV on different read coverage. We generated five different coverage (10×, 15×, 20×, 25× and 30×) by down-sampling of 150 bp WGS reads of HG002. As shown in Supplementary Figure S5, ClipSV identified 5019 deletions and 4130 insertions with the sequencing reads of 30× coverage. Among them, 3973 (79.2%) deletions and 3410 (82.6%) insertions could be supported by CCS reads. When read coverage was decreased to 15×, ClipSV identified 4434 deletions and 3962 insertions. Among them, 3475 (78.4%) deletions and 2831 (71.5%) insertions could be supported by CCS reads. Therefore, compared with 30× read coverage, ClipSV could retain 87.5% (3475/3973) sensitivity for deletions and 83.0% (2831/3410) sensitivity for insertions with 15× read coverage. When the read coverage was down-sampled to 10×, ClipSV could retain 72.2% (2868/3973) sensitivity for deletions and 60.0% (2045/3410) sensitivity for insertions.

**Table 1.** Run time and memory usage for the SV tools

	2 × 150 bp WGS (30×)			2 × 250 bp WGS (30×)		
Tools	ClipSV	Lumpy	Manta	ClipSV	Lumpy	Manta
Wall-clock (hours)	2.9	5.3	0.4	3.5	6.7	1.0
Memory (GB)	22.3	14.7	1.3	29.3	22.9	1.5

We also compared the running time and memory cost of the three tools (Table 1). With a high-performance cluster with 12 threads, the three tools could be finished within 7 h for one WGS sample with a 30× sequencing depth. The peak memory costs were within 30 GB for these tools. Among them, Manta had the best performance in both running time and memory cost. Compared with Lumpy, ClipSV had a shorter running time but a higher memory cost. The 250 bp sequencing reads required more computational resources for all the three tools. Based on overall performance, ClipSV has a potential to serve as a widely used tool for SV analysis in individual genomes as well as large consortium cohorts.

## Supplementary Material

lqab003_Supplemental_File

## Data Availability

Implemented in Python, ClipSV is freely available at https://github.com/ChongLab/ClipSV,

## Notes

Present address: Peng Xu, Department of Genetics & Genomic Sciences, Icahn School of Medicine at Mount Sinai, New York, NY 10029, USA.
