# Supplementary material for: ClipSV: improving structural variation detection by read extension, spliced alignment and tree-based decision rules
Source: NAR Genom Bioinform. 2021 Feb 1;3(1):lqab003. doi: 10.1093/nargab/lqab003 (PMC7850140; doi:10.1093/nargab/lqab003)
Supplement: lqab003_Supplemental_File [file lqab003_supplemental_file.docx]

| **Supplementary Table S1.** Performance of SV tools by simulation (2x150bp 30X) | | | |
| --- | --- | --- | --- |
|  | Lumpy | Manta | ClipSV |
| **Deletion** | **2620 (2638)** | **3757 (3759)** | **3745 (3787)** |
| Sensitivity | 50.5% | 72.4% | 72.1% |
| Precision | 99.3% | 99.9% | 98.9% |
| **Insertion** | **0 (76)** | **5737 (5752)** | **6096 (6164)** |
| Sensitivity | 0.0% | 83.7% | 88.9% |
| Precision | 0.0% | 99.7% | 98.9% |
| **Inversion** | **190 (190)** | **181 (182)** | **180 (182)** |
| Sensitivity | 95.0% | 90.5% | 90.0% |
| Precision | 100.0% | 99.5% | 98.9% |
| **Translocation** | **116 (1309)** | **116 (128)** | **107 (110)** |
| Sensitivity | 96.7% | 96.7% | 89.2% |
| Precision | 8.9% | 90.6% | 97.3% |
| **Total** | **2926 (4213)** | **9791 (9821)** | **10128 (10243)** |
| Sensitivity | 23.7% | 79.2% | 81.9% |
| Precision | 69.5% | 99.7% | 98.9% |
| **F1-score** | **0.353** | **0.883** | **0.896** |

| **Supplementary Table S2.** Performance of svABA by simulation (30X) | | |
| --- | --- | --- |
|  | 2x150 bp | 2x250 bp |
| BND overlapping with deletion | 1389 (26.8%) | 1136 (21.9%) |
| BND overlapping with insertion | 1332 (19.4%) | 1130 (16.5%) |
| BND overlapping with inversion | 200 (100%) | 200 (100%) |
| BND overlapping with translocation | 120 (100%) | 120 (100%) |
| Total detected events | 3041 | 2586 |
| Total BND | 10376 | 8657 |
| Sensitivity | 24.6% | 24.6% |
| Precision | 29.3% | 35.1% |
| F1-score | 0.267 | 0.289 |

| **Supplementary Table S3.** Performance of SV tools by simulation (2x250bp 30X) | | | |
| --- | --- | --- | --- |
|  | Lumpy | Manta | ClipSV |
| **Deletion** | **2486 (2542)** | **4049 (4064)** | **4483 (4588)** |
| Sensitivity | 47.9% | 77.4% | 86.3% |
| Precision | 97.8% | 99.6% | 97.7% |
| **Insertion** | **0 (83)** | **6128 (6151)** | **6383 (6525)** |
| Sensitivity | 0.0% | 89.4% | 93.1% |
| Precision | 0.0% | 99.6% | 97.8% |
| **Inversion** | **187 (187)** | **182 (187)** | **183 (188)** |
| Sensitivity | 93.5% | 91.0% | 91.5% |
| Precision | 100.0% | 97.3% | 97.3% |
| **Translocation** | **112 (1750)** | **116 (162)** | **112 (115)** |
| Sensitivity | 93.3% | 96.7% | 93.3% |
| Precision | 6.4% | 71.6% | 97.4% |
| **Total** | **2785 (4562)** | **10475 (10564)** | **11161 (11416)** |
| Sensitivity | 22.5% | 84.7% | 90.2% |
| Precision | 61.0% | 99.2% | 97.8% |
| **F1-score** | **0.329** | **0.914** | **0.939** |

**
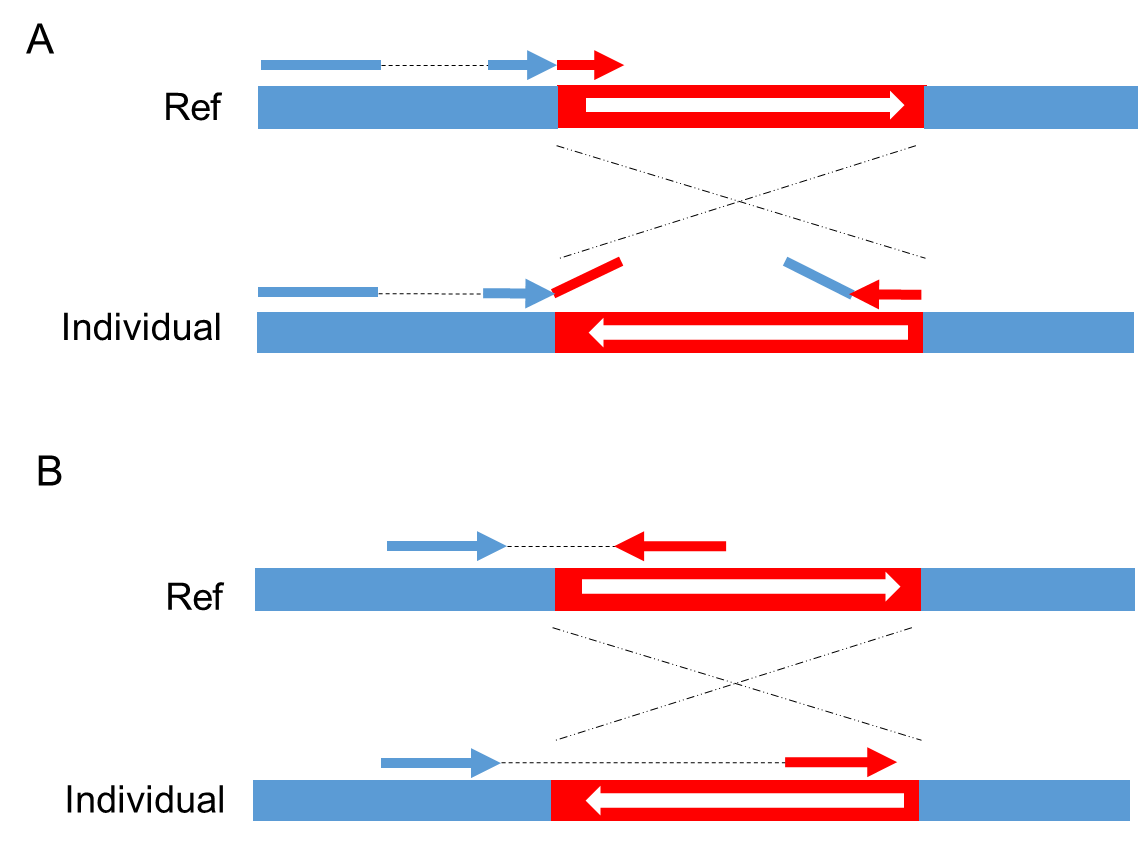
**

**Supplementary Figure S1.** Schematic diagram of inversion detection by split reads (A) and discordant reads pairs (B). For split reads, if the two alignments have opposite orientations, an inversion spanning the two breakpoints will be identified. For discordant read pairs, if the orientations of the aligned read pairs are the same, this read pair will support a large inversion event.

**
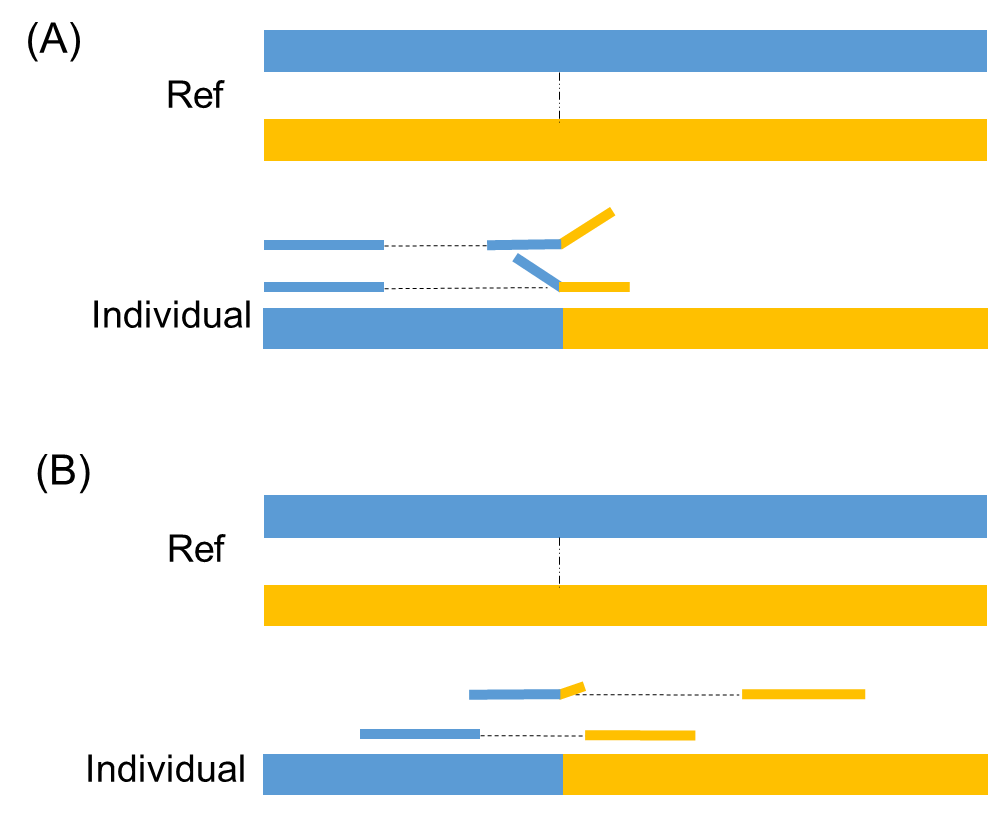
**

**Supplementary Figure S2.** Schematic diagram of translocation detection by split reads (A) and discordant read pairs (B). For split reads, the breakpoints of the two alignments are regarded as the translocation site. For discordant read pairs, the translocation sites are approximated by the alignments of the two read pairs.

**
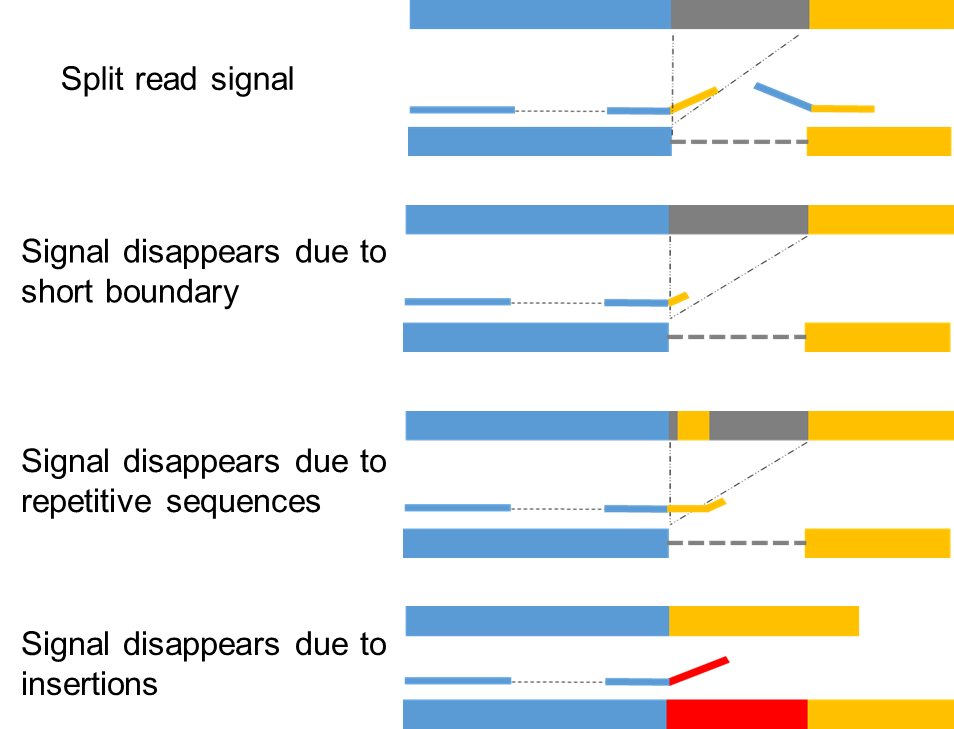
**

**Supplementary Figure S3.** The scenarios where clipped reads can rescue SV events missed by split reads. The first row shows a typical scenario for split reads to detect a deletion. The following part shows several cases which only have clipped reads but no split reads. The blue and yellow color indicate the upstream and downstream sequences, respectively. The grey and red colors indicate the deletion and insertion region, respectively. The yellow color with grey region indicates the deleted sequences are similar with the downstream sequences.


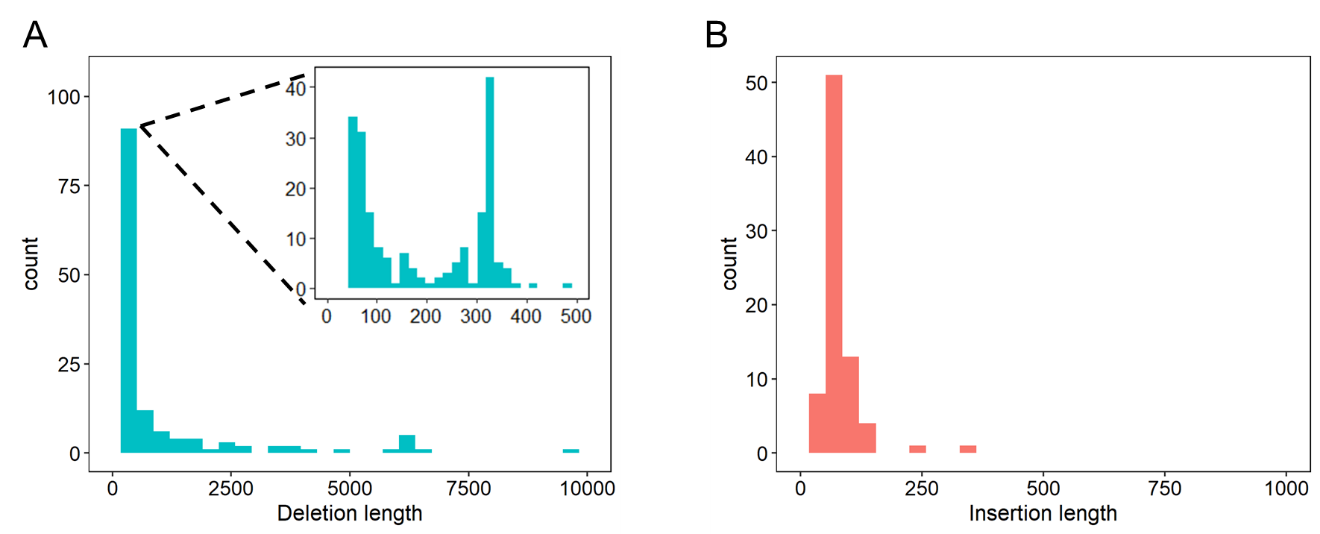


**Supplementary Figure S4.** Length distribution of SV events detected by splice alignment and read extension on HG002 chromosome 1. (A) Length distribution of deletions less than 10 kb. Two peaks around 300 bp and 6000 bp correspond to the SVs caused by Alu and LINE retrotransposons, respectively. The inset showed the distribution of deletions less than 500 bp. (B) Length distribution of insertions less than 1 kb. The splice alignment and read extension method only detects short insertions. The larger events will be identified by the local assembly method in ClipSV.


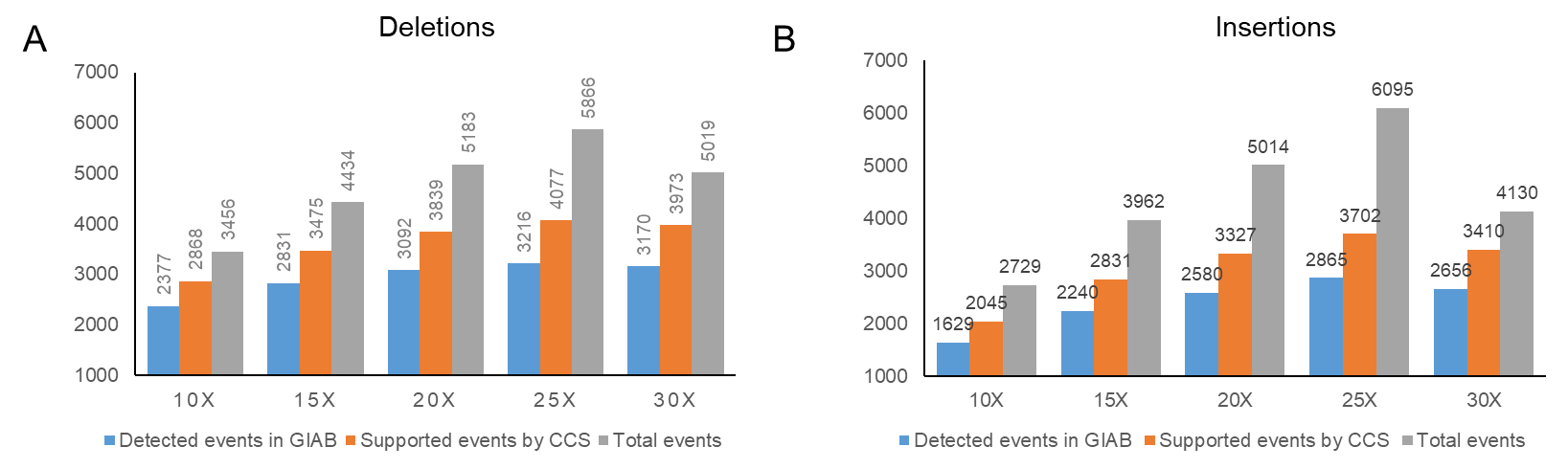


**Supplementary Figure S5.** Performance of ClipSV on different sequencing depths. Barplots show the performance of ClipSV on deletion (A) and insertion (B) detection with different read depths. The HG002 sample with 2x150 bp sequencing reads were down-sampled to different sequencing depths and then used for evaluation. SV events detected by ClipSV were compared with GIAB high-confident callset and CCS results, respectively.
